# Characterization of the complete chloroplast genome of the prickly blue poppy *Meconopsis horridula* Hook. f. & Thomson (Ranunculales: Papaveraceae)

**DOI:** 10.1080/23802359.2021.1882902

**Published:** 2021-03-11

**Authors:** Quehu Dan, Qien Li, XianJia Li, Renqian Suonan, Duojie Dongzhi, Xiao Guo

**Affiliations:** a Tibetan Medicine Research Center, Tibetan Medical College, Qinghai University, Xining, PR China; b State Key Laboratory of Tibetan Medicine Research and Development, Xining, PR China; c Tibetan Medical Hospital of Qinghai Province, Xining, PR China

**Keywords:** Iterative mapping, MITObim, phylogenetic analysis, plastid genome, prickly blue poppy

## Abstract

The prickly blue poppy (*Meconopsis horridula* Hook. f. & Thomson) is a traditional Tibetan medicinal herb with high values. In this study, its chloroplast genome was determined to be 153,761 bp in length with an A + T-biased base composition, and comprises a pair of inverted repeat (IR) regions (26,030 bp), separated by a large single-copy (LSC) region (83,803 bp) and a small single-copy (SSC) region (17,898 bp). A total of 113 gene species were annotated, with 20 of them being completely or partially duplicated and 18 of them harboring one or two introns. Phylogenetic analysis suggests that *M. horridula* is closely related to *Meconopsis racemosa* Maxim.

The prickly blue poppy (*Meconopsis horridula* Hook. f. & Thomson) is a perennial herb within the family Papaveraceae (order Ranunculales), and is mainly distributed in western China (incl. Gansu, Qinghai, Sichuan, Yunnan, and Tibet), Bhutan, northeastern India, northern Myanmar and Nepal with an elevation of 3600–5400 m (Zhang and Christopher 2008; Zhao Y et al. 2010). As a traditional Tibetan medicine, this herb has been used to promote blood circulation, remove bruises and stasis, and relieve chest pain (Zhao F et al. 2020), and may play a potential role in cancer treatment (Fan et al. 2015; Tang et al. 2020). To date, most studies of *M. horridula* have been focused upon its phytochemistry (Takeda et al. 1996; Xie et al. 2001; Wu et al. 2009; Guo et al. 2014; Liu et al. 2014; Zhao F et al. 2020). In this study, its complete chloroplast genome was retrieved from Illumina sequencing data. In addition, its placement within the family Papaveraceae was also investigated by phylogenetic analysis.

Fresh leaves of a single individual of *M. horridula* were collected from NagunLha Mountain (30°59′21′′N, 91°11′13′′E) with the voucher specimen deposited at Qinghai University (https://www.qhu.edu.cn/; Qien Li, qienli@qhu.edu.cn) under the voucher number LQE-2019-068. Total genomic DNA was isolated with the DNeasy Plant Mini Kit (Qiagen, San Diego, CA). DNA sequencing was conducted on the Illumina HiSeq X Ten Sequencing System (Illumina, San Diego, CA), and a total of 73.88 M of 150-bp paired raw reads were obtained. Assembly of the chloroplast genome was done using MITObim version 1.9 (Hahn et al. 2013) with that of *Meconopsis racemosa* Maxim. (GenBank accession no.: MH394402) (Zeng et al. 2018) as the initial reference. Genomic annotation was done by aligning with those of its confamilial counterparts, e.g. *Meconopsis punicea* Maxim. (MK533648) (Li X et al. 2019), *Papaver rhoeas* L. (MF943221), and *Papaver orientale* L. (MF943222) (Zhou et al. 2018).

The chloroplast genome of *M. horridula* was determined to be 153,761 bp in length, and comprises a pair of inverted repeat (IR) regions (26,030 bp), separated by a large single-copy (LSC) region (83,803 bp) and a small single-copy (SSC) region (17,898 bp). The base composition is asymmetric with an overall A + T content of 61.3% (‘light strand’). Relatively, the A + T content is obviously higher in the SSC region (66.9%) than in the LSC region (62.8%) or the IR regions (57.0%). A panel of 113 gene species was annotated, including 79 protein-coding gene (PCG), 30 *tRNA*, and four *rRNA* gene species. Twenty gene species are completely or partially duplicated, including nine PCGs (*ndh*B, *rpl*2, *rpl*23, *rps*7, *rps*12, *rps*19, *ycf*1, *ycf*2, and *ycf*15), seven tRNAs (*trn*A-UGC, *trn*I-CAU, *trn*I-GAU, *trn*L-CAA, *trn*N-GUU, *trn*R-ACG, and *trn*V-GAC) and all four rRNAs (*rrn*4.5, *rrn*5, *rrn*16, and *rrn*23). The presence of a single intron was detected in 16 gene species (*atp*F, *ndh*A, *ndh*B, *pet*B, and *pet*D, *rpl*2, *rpl*16, *rpo*C1, *rps*12, *rps*16, *trn*A-UGC, *trn*G-UCC, *trn*I-GAU, *trn*K-UUU, *trn*L-UAA, and *trn*V-UAC), and the presence of double introns in two gene species (*clp*P and *ycf*3).

A maximum-likelihood phylogeny was reconstructed using chloroplast PCGs for a total of 12 taxa with available chloroplast genomes within the family Papaveraceae with the program MEGA version 7 (Kumar et al. 2016) (Figure 1). ‘GTR + G+I’ was employed as the best-fit nucleotide substitution as suggested by MEGA version 7. The outgroup taxa used in this study are two species from the family Lardizabalaceae (order Ranunculales), i.e. *Akebia quinata* (Houtt.) Decne. (KX611091) (Li B et al. 2016) and *Decaisnea insignis* (Griff.) Hook.f. & Thomson (KY200671) (Li B et al. 2017). As expected, all four taxa within the genus *Meconopsis* (incl. *M. horridula*, *M. racemose*, *Meconopsis integrifolia* (Maxim.) Franch. and *Meconopsis punicea* (Maxim.) were clustered together. This also holds true for the three taxa within the genus *Papaver* (incl. *P. orientale*, *P. rhoeas*, and *Papaver somniferum* L.). *M. horridula* was found to be most closely related to *M. racemosa*.

**Figure 1.**
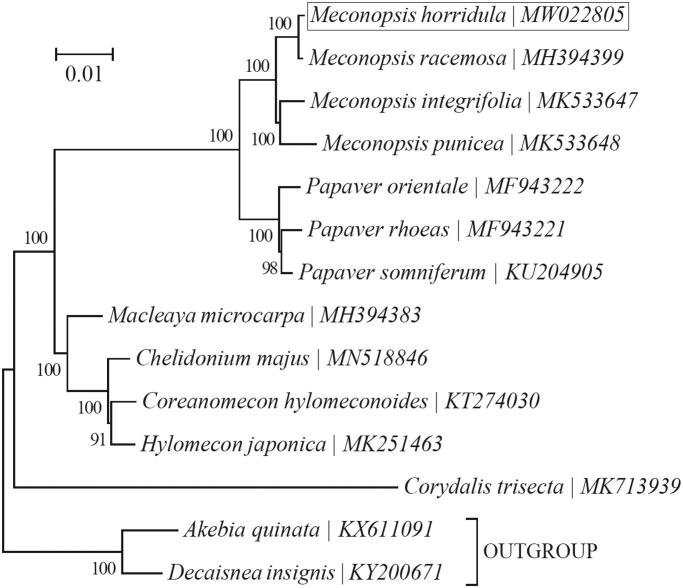
Phylogeny of the family Papaveraceae based on the maximum-likelihood analysis of the concatenated coding sequences of chloroplast PCGs. The best-fit nucleotide substitution model is ‘GTR + G+I’. The bootstrap values are based on 100 random replicates. Two species within the family Lardizabalaceae were included as outgroup taxa.

## Data Availability

The genome sequence data that support the findings of this study are openly available in GenBank of NCBI at (https://www.ncbi.nlm.nih.gov/) under the accession no. MW022805. The associated BioProject, SRA, and Bio-Sample numbers are PRJNA689716, SRR13357474, and SAMN17215169, respectively.
